# Treatment resistant hypertension among ambulatory hypertensive patients: A cross sectional study

**DOI:** 10.1371/journal.pone.0232254

**Published:** 2020-04-28

**Authors:** Solomon Weldegebreal Asgedom, Kidus Amanuel, Meles Tekie Gidey, Yirga Legesse Niriayo, Kidu Gidey, Tesfay Mehari Atey

**Affiliations:** 1 Clinical Pharmacy Unit, School of Pharmacy, College of Health Sciences, Mekelle University, Mekelle, Tigray, Ethiopia; 2 Social Pharmacy and Pharmaco-Epidemiology Unit, School of Pharmacy, College of Health Sciences, Mekelle University, Ethiopia; International University of Health and Welfare, School of Medicine, JAPAN

## Abstract

**Background:**

Treatment resistant hypertension(TRH) is detrimental risk of cardiovascular and premature deaths. Globally, the prevalence of resistant hypertension is inclining from time to time and it is yet to be determined in Ethiopia.

**Objective:**

To assess the prevalence of apparent TRH and its predictors among ambulatory hypertensive patients on follow up in hypertension clinic of Mekelle Hospital, Northern Ethiopia.

**Method:**

A hospital based cross sectional study was conducted from Nov 25, 2018 to July 20, 2019, among 338 adult ambulatory hypertensive patients on follow up in Mekelle Hospital hypertension clinic. Hypertensive patient aged ≥18 years who were on regular follow up and taking antihypertensive medications for at least 6 months were included in the study. A simple random sampling technique was used to recruit the study patients.

**Results:**

A total of 338 adult ambulatory hypertensive patients were analysed. More than half, 182 (53.8%) patients were females and the average age of the patients was 58.9 ±11.5. Three hundred thirty-three (98.5%) patients had no family history of hypertension. Majority, 66.8% of the patients were on monotherapy. The prevalence of apparent TRH was calculated to be 8.6% [Confidence Interval = 0.056–0.116]. Patients with Body Mass Index(BMI) greater than 30[Adjusted Odds Ratio(AOR) = 12.1, 95%CI:2.00–73.19, p = 0.007] and longer duration of hypertension were the predictors of resistant hypertension.

**Conclusion:**

Even if escalation of antihypertensive medications was not aggressive, apparent TRH was common in the study setting. Obesity (BMI greater than 30) and longer duration of hypertension since diagnosis were the predictors of TRH. Meticulous emphasis should be placed on to detect the prevalence of true hypertension resistance and future studies should discover the impact of aggressive antihypertensive medications scale up on the risks of TRH.

## Background

Hypertension(HTN) and its consequences are a major global public health problem, affecting greater than one fourth of adults in developed societies [1]. It is the leading cause of premature death from preventable medical illness worldwide [2]. Appropriate management and sustainable blood pressure(BP) control of hypertension are indispensable for obviation of organ damage and cardiovascular consequences [3]. Globally controlling BP of hypertensive patients is challenging. In 2010 the level of BP control in a study done from 90 countries was 7.7% [2]. Worldwide, hypertensive patients with uncontrolled BP reached about one billion [4]. Often, in Sub-Saharan Africa countries patients who achieve target BP control are less than 30% [5]. In Ethiopia the proportion of hypertensive patients who have controlled BP vary from 30.1%-50.4% [6–11]. Some few patients have refractory uncontrolled BP to dual and triple regimen antihypertensive therapy [12].

The global prevalence of treatment resistance hypertension(TRH) is significantly heterogeneous. A global meta-analysis study illustrated a 10.3% prevalence of true resistant hypertension [13]. A United States of America study also found an apparent treatment resistance hypertension (aTRH) of 17.7% based on 2008 definition and 19.7% according to 2018 Scientific Statement Definitions[14]. Antihypertensive and Lipid‐Lowering Treatment to Prevent Heart Attack Trial (ALLHAT) study also classified 15% of the studied patients to have resistant hypertension [15]. The highest (38%) prevalence of resistant hypertension was reported from a study in Florida [16]. In Africa TRH prevalence was reported from Cameroon[17], Burkina Faso[18], Lesotho[19] and Algeria[20] with prevalence’s of 11.7%, 14.6%, 14.3% and 19.0%, respectively and a meta-analysis of the studies found a pooled prevalence of 12.1% (95% CI: 8.0% to 17.7%) [21]. Worldwide, prevalence and incidence of resistant hypertension is increasing on the rudimentary regimen when compared to previous thoughts [22, 23]

There are many factors identified to be associated with incidence and prevalence of resistance hypertension. Studies showed that diabetes mellitus[16, 24–26], history of cardiovascular disease[16], longer hypertension duration[16], left ventricular hypertrophy[16], heart failure[16] glomerular filtration rate [25, 27, 28] and black race [24] had been significantly associated with TRH.

In our country Ethiopia TRH prevalence is not yet determined. Knowing, detecting and determining the prevalence of TRH and its predictors will be conducive for health care professionals to draft strategies to solve the clinical problem. Therefore, the main aim of this study was to determine prevalence of apparent TRH and its predictors in Mekelle Hospital, Northern Ethiopia.

## Methods

### Study area and period

The study was conducted in Mekelle Hospital at outpatient hypertension follow up clinic. Mekelle Hospital is found at Mekelle city, the capital of Tigray region. It if found 783Km away from the capital city of Ethiopia, Addis Ababa. It is found in the northern part of the country. In the city there are four governmental organizations, one nongovernmental organization(NGO) hospital and nine health centers. Hypertension follow up medical and pharmaceutical cares for ambulatory patients is delivered in Mekelle Hospital and Ayder Comprehensive Specialized Hospital(ACSH). Mekelle hospital was selected for the study conduction randomly using lottery method. The study was conducted from Nov 25, 2018 to July 20, 2019.

### Study design and population

The study design used to conduct the study was a cross sectional study design. Adult ambulatory hypertensive patients having regular follow up at Mekelle Hospital hypertensive clinic were the source of population for the study. The study population was all adult ambulatory hypertensive patients who had regular follow-up at the hypertensive clinic and those who fulfil the inclusion criteria’s. Patient’s medical registry was the source of data for the study and sociodemographic characteristics, family history of hypertension, BP measurements, Body Mass Index (BMI), type of antihypertensive medications prescribed, comorbidity, laboratory results, complications, duration of hypertension since diagnosis, aspirin use and statins use were the variables collected from the patients’ medical charts. Patients who at least took one antihypertensive medication for greater than 6 months were included in the study. On the other side, patients who had incomplete medical registration information such as patients with no record of full BP measurements, BMI and type of antihypertensive medications used and other variables, and patients on dietary approach to stop hypertension (DASH) were excluded from the study.

Single population proportion formula was used to determine the sample size with the assumption of 95% confidence level, 5% margin of error and the apparent TRH prevalence rate of 50%. A total of about 2000 hypertensive patients were registered to have regular follow up in the hypertension clinic in Mekelle Hospital. By adjusting this using finite population correction formula the minimum sample size was found to be 338. A systematic random sampling technique was used to enroll the patients on the study. During the period of data collection, a total of 761 hypertensive patients fulfilled the inclusion criteria and did not fulfilled the exclusion criteria’s, thus computed for lottery method. The patient’s registration card number was assigned a number and every second patient was involved in the data collection process. The trend of patient’s selection for our study is depicted in S1 Fig.

### Study variables

Age, BMI, diagnosis, family history of hypertension, duration of hypertension since diagnosis, residence, complications, angiotensin converting enzyme inhibitors (ACEIs) use, calcium channel blockers use, thiazide diuretics use, concomitant medications use (aspirin use, statins use) and comorbidity were the independent variables. Meanwhile, apparent TRH was our primary outcome.

### Data collection procedure

Firstly, and for most, data collection tool was developed by reviewing reputable published literatures [24, 29, 30] and taking patients’ medical registry format of the hospital in to consideration. The data collection tool was pretested on 33 hypertensive patients. Pretest was done on the patients’ medical registries in the ambulatory follow up clinic. The patients who were pretested were excluded from data analysis. According to the findings of the pretest amendment and modification of the data collection format was done. A format redaction and a variables scale up modification was deployed in the data collection format.

Prior to data collection, the study was approved through Ethical Review Board of Mekelle University, College of Health Sciences, School of pharmacy. Letter of permission and support request was sent from the university to Mekelle Hospital. The hospital also offered us a letter of permission to the clinic. Before data collection an oral consent was informed to the patients. The data collection tool was also entirely confidential. Patients name, and address were not collected to keep patient’s confidentiality during data collection. Two registered pharmacists working outside the hospital were recruited to collect the data and they were trained before they started data collection. The principal investigator strictly followed the data collection process and procedure every day.

### Data analysis

After data collection, the quality of data was checked and assured for its reliability, completeness, consistence and was finally cleaned. Quality of the data was assured through double entry method. That is, the two data collectors entered their data and exchange each other and recheck for any error detection. To minimize mistakes, we also checked, occurrence of missing values, minimum and maximum values of the continuous variables. Moreover, we also checked any deviation of labelled valued on some selected cases between the entered value and the data collected from patients. Until the data analysis and writing up was completed the data was handled with the principal investigator. The data was entered in to SPSS version 22 for analysis. Before we computed logistic regression analysis and prior to estimation of means and SD of the continuous variables, we checked normality distribution of all the variables using skewness, kurtosis and q-q plots. All the variables were approximately normally distributed. We did bivariate logistic regression to identify factors associated with apparent TRH then six variables were included in the multivariable analysis. All the variables supposed to be involved in the multivariable regression were checked for model fitness using ominous tests of coefficients fitness analysis model. To assess collinearity of the variables each other a variance inflation factor(VIF) was determined. To identify aTRH predictors a multiple logistic regression analysis was used. Level of statistical significance was declared at a p value of less than 0.05 for all types of analysis.

### Operational definition

Six months’ patients’ BP measurements were collected, reviewed and the last follow up BP measurement was used to declare BP status of the patients, and it was also used to declare treatment resistance in patients taking three antihypertensive medications without regimen change. When patients changed antihypertensive regimen the last consecutive follow-up months BP measurements were used to affirm treatment resistance. TRH was explained as unable to meet the goal BP of <140/90 mm Hg to 3 different antihypertensive medications at their maximum dosages, one of them must be a diuretic. Patients who had controlled BP (<140/90 mmHg) with 4 and above different antihypertensive medications were considered to be resistant to treatment [31]. The type of resistant hypertension in our study was apparent TRH since pseudo resistance hypertension was not excluded using 24-h ambulatory BP monitoring, proper office BP measurement techniques and confirmation of medication adherence through pill counts. BMI was calculated by dividing the patients weight to height^2^. Comorbidity was defined as a medical disease or condition simultaneously existed with hypertension independent of hypertension disease. Complication is a secondary medical condition or disease which developed in the courses of primary hypertension condition.

## Results

In our study a total of 338 adult ambulatory hypertensive patients were enrolled in the study and were analyzed. From the total patients enrolled, when 182(53.8%) patients were females, 156(46.2%) patients were men. The average age (Mean ± SD) of the patients was 58.9 ±11.5 years. Thirty-five (10.4%) hypertensive patients were classified in the age category of less than 40 years old while 106(31.4%) were in the age of 41–55 years old. The average duration of hypertension since diagnosis was 5.1±2.3 years which ranged from a minimum of 1 year to a maximum of 23 years. More than half, 56.8%, of the studied patients had a normal BMI (18.5–24.9kg/m^2^). The mean of BMI (Mean ± SD) of the analyzed patients was 22.72 ±9.13kg/m^2^. Regarding clinical features of the patients, 245(72.5%) hypertensive patients had evidence of complications and most, 321(95%) patients had no evidence of comorbidity. The mean SBP of the patients was 150.3±21.8mmHg and the average DBP of the hypertensive patients in the entire follow us was 87.9±24.0mmHg. The average SBP and DBP measurements of the non-resistant hypertensive patients was 149.8±87.7 and 87.7±24.9mmHg respectively. Moreover, TRH patient had a mean SBP and DBP of 154.9±20.2 and 90.3±12.7 respectively. When 61.5% of the non-resistant patients had uncontrolled BP, all the patients (8.6%) who had apparent TRH had uncontrolled BP. Overall, 70.1% of the patients studied had uncontrolled BP [Table 1].

**Table 1:** Socio-demographic and clinical characteristics of adult ambulatory hypertensive patients in Mekelle Hospital, 2019.

Variables		Number(%)
Sex	Male	156(46.2)
	Female	182(53.8)
Age(in years)	≤40	35(10.4)
	41–55	106(31.4)
	56–70	147(43.5)
	≥71	50(14.8)
Residence	Urban	312(92.3)
	Rural	26(7.7)
BMI(kg/m^2^)	≤18.5	50(14.5)
	18.5–24.9	192(56.8)
	25.0–29.9	91(26.9)
	≥30.0	5(1.5)
Diagnosis	HTN	321(94.9)
	HTN+DM	8(2.4)
	HTN + Thyrotoxicosis	2(0.6)
	HTN + CHF	6(1.8)
	HTN + RVI	1(0.3)
HTN duration since diagnosis	<6 years	226(66.9)
	6–23 years	112(33.1)
Family history of HTN	Yes	5(1.5)
	No	333(98.5)
Complication	Yes	245(72.5)
	No	93(27.5)
ACEI use	Yes	128(37.9)
	No	210(62.1)
HCT use	Yes	249(73.7)
	No	89(26.3)
CCB use	Yes	102(30.2)
	No	236(69.8)
Aspirin use	Yes	9(2.7)
	No	329(97.3)
Statin use	Yes	1(0.3)
	No	337(99.7)
Frequency of Anti HTN drug change	No change	199(58.9)
	Once	77(22.8)
	Twice	62(18.3)
Comorbidity	Yes	17(5.0)
	No	321(95.0)

ACEI: Angiotensin Converting Enzyme Inhibitor, HCT: Hydrochlorothiazide, CCB: Calcium Channel Blocker, HTN: Hypertension, RVI: Retroviral Infection, CHF: Congestive Heart Failure, DM: Diabetes Mellitus.

**Fig 1:**
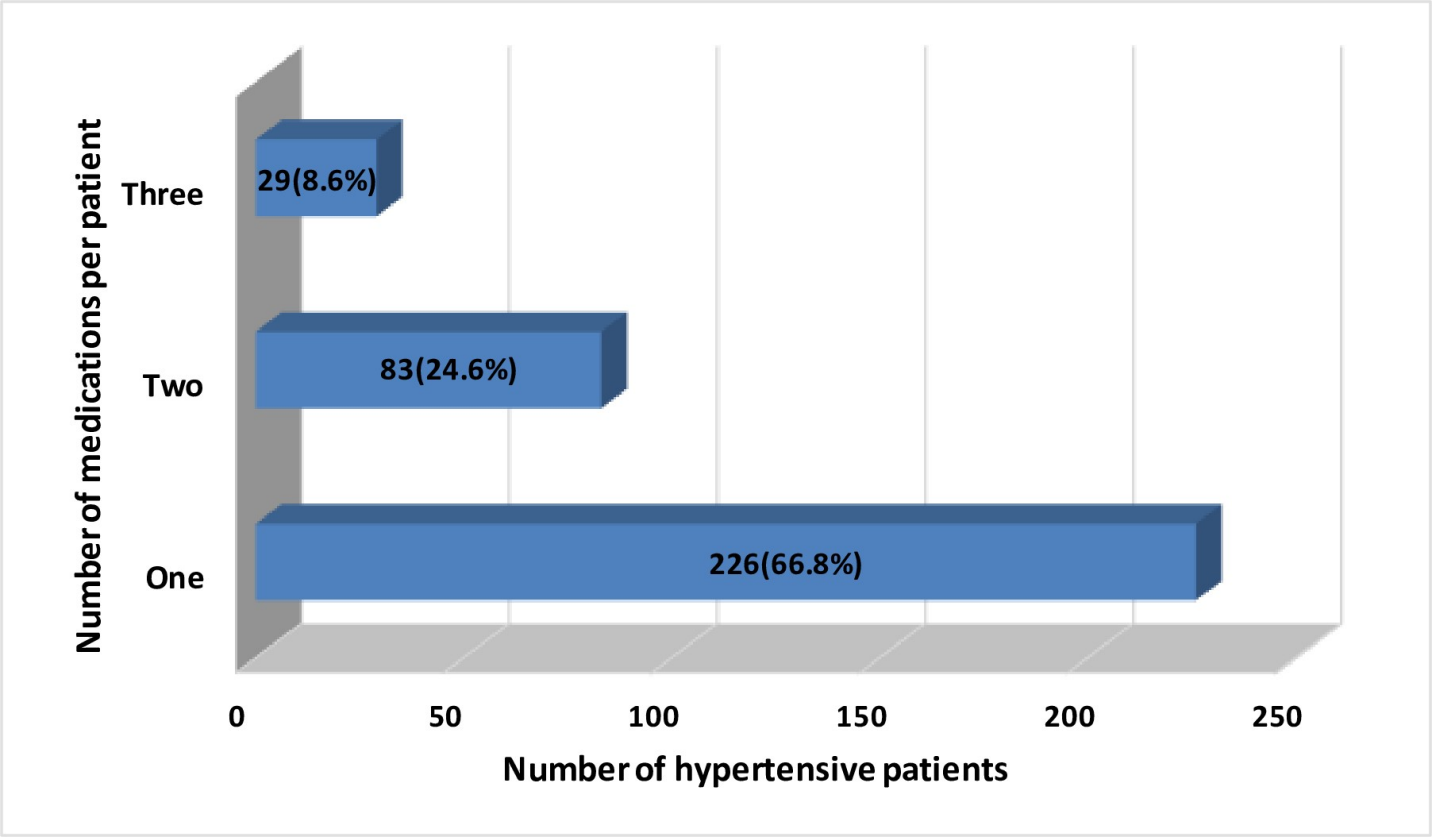
Number of antihypertensive medications used among adult ambulatory hypertensive patients in Mekelle Hospital, 2019.

Among the total hypertensive patients studied, 226(66.8%) were on monotherapy and 29(8.6%) were on triple antihypertensive regimen [Fig 1]. Hydrochlorothiazide (HCT) monotherapy was the most commonly prescribed antihypertensive medication which was given for 153(45.3%) patients. For those patients who took combination antihypertensive medications, HCT + Enalapril was the most commonly prescribed regimen given for 46(13.6%) patients followed by HCT + Enalapril + Nifedipine which was prescribed for 29(8.6%) patients [Table 2].

**Table 2:** Type of antihypertensive medications and regimens used among adult ambulatory hypertensive patients in Mekelle Hospital, 2019.

Type of antihypertensive medication	Frequency(%)
HCT	153(45.3)
HCT + Enalapril	46(13.6)
Enalapril	37(11)
Nifedipine	32(9.5)
HCT + Enalapril + Nifedipine	29(8.6)
HCT +Nifedipine	21(6.2)
Enalapril + Nifedipine	12(3.6)
Amlodipine	4(1.2)
Enalapril + Amlodipine	4(1.2)

HCT: Hydrochlorothiazide

**Fig 2:**
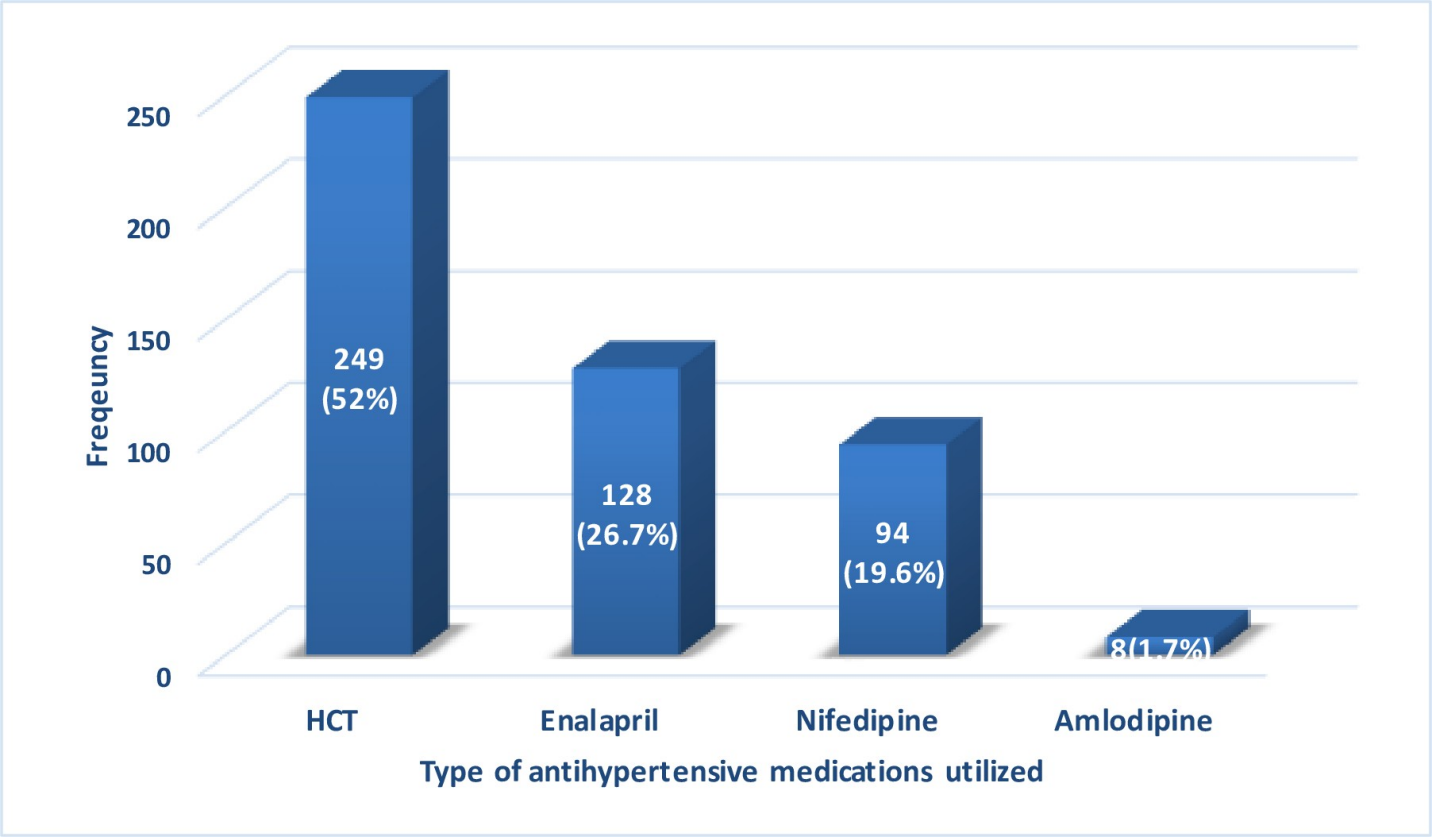
Types of antihypertensive medications frequently utilized among adult ambulatory hypertensive patients in Mekelle Hospital, 2019. HCT: Hydrochlorothiazide.

Concerning frequency of antihypertensive medications prescribed, HCT was the most frequently prescribed medication either in monotherapy or combination therapy. It was prescribed for 52% of the total 479 medical order frequencies [Fig 2].

Apparent TRH was detected in 8.6%[CI:0.056–0.116] of the studied patients. On univariate logistic regression analysis BMI greater than 30 [Crude Odds Ratio(COR) = 20, 95%CI:3.56–112.3, P = 0.001] was significantly associated with TRH as compared to patients with BMI less than 18.5kg/m^2^. Moreover, patients with longer duration of hypertension (who were on treatment for 6 years and above) [COR = 4.41, 95%CI:1.98–5.6, p = 0.000] were 4 times more likely to be resistant for treatment as compared to patients who were on treatment for less than 6 years (Table 3).

**Table 3:** Univariate logistic regression analysis of factors associated with treatment resistant hypertension among adult ambulatory hypertensive patients in Mekelle Hospital, 2019.

Variable	TRH		P-value	COR (95%CI)
	No	Yes		
**Sex**				
Male	142(42%)	14(4.1%)	0.811	1.1(0.51–2.35)
Female	167(49.4%)	15(4.4%)	1	1
**Age**				
<41	31(10.0%)	4(13.8%)	0.833	1.161(0.289–4.672)
41–55	100(32.4%)	6(20.7%)	0.329	0.540(0.157–1.862)
56–71	133(43.0%)	14(48.3%)	0.922	0.947(0.323–2.777)
> = 71	45(14.6%)	5(17.2%)	1	1
**BMI**				
**<**18.5	40(12.9%)	3(10.3%)	1	1
18.5–24.5	182(58.9%)	8(27.6%)	0.445	0.586(0.149–2.307)
24.6–29.9	83(26.9%)	12(41.4%)	0.330	1.928(0.515–7.218)
>30	4(1.3%)	6(20.7%)	0.001	20.00(3.562–112.299)
**Complication**				
No	87(27.2%)	6(20.7%)	1	1
Yes	222(71.8%)	23(79.3%)	0.392	0.67(0.26–1.69)
**Comorbidity**				
Yes	22(7.1%)	1(3.4%)	1	1
No	287(92.9%)	28(96.6%)	0.65	0.68(0.08–5.12)
**HTN duration since diagnosis**				
<6 years	216(63.9%)	10(3%)	1	1
≥6 years	93(27.5%)	19(5.6%)	0.000	4.41(1.98–5.6)

BMI: Body Mass index, TRH: Treatment Resistant Hypertension, COR: Crude Odds Ratio, HTN: Hypertension, CI: Confidence Interval

Before we run multivariable logistic regression, bivariable logistic regression was done and six variables were included in the multivariable logistic regression analysis. The model containing all predictors deployed in the multivariable logistic regression was run and it was statistically significant (Chi-square = 141.79, df = 102, P = 0.006). Collinearity of the variables was also conducted and the VIF value of all the variables ranged from 1.06–1.56 which indicates that the variables were not collinear. Patients with BMI greater than 30[AOR = 12.1, 95%CI:2.00–73.19, p = 0.007] and patients on treatment for 6 years and above [AOR = 4.1, 95%CI:1.74–9.57, p = 0.001] were found to be the predictors of TRH (Table 4).

**Table 4:** Multivariate logistic regression analysis of factors associated with treatment resistant hypertension among adult ambulatory hypertensive patients in Mekelle Hospital, 2019.

Variable	TRH		P-value	AOR (95%CI)
	No	Yes		
**Sex**				
Male	142(42%)	14(4.1%)	0.72	1.19(0.46–3.1)
Female	167(49.4%)	15(4.4%)	1	1
**Age in years**				
<41	31(10.0%)	4(13.8%)	0.46	1.98(0.33–11.86)
41–55	100(32.4%)	6(20.7%)	0.67	0.75(0.19–2.94)
56–71	133(43.0%)	14(48.3%)	0.59	1.41(0.41–4.89)
> = 71	45(14.6%)	5(17.2%)	1	1
**Duration of HTN since diagnosis**				
<6 years	216(63.9%)	10(3%)	1	1
≥6 years	93(27.5%)	19(5.6%)	0.001	4.1(1.74–9.57)
**Complication**				
No	87(27.2%)	6(20.7%)	1	1
Yes	222(71.8%)	23(79.3%)	0.27	0.51(0.15–1.67)
**Comorbidity**				
Yes	22(7.1%)	1(3.4%)	1	1
No	287(92.9%)	28(96.6%)	0.37	0.32(0.03–3.94)
**BMI**				
**<**18.5kg/m^2^	40(12.9%)	3(10.3%)	1	1
18.5–24.5kg/m^2^	182(58.9%)	8(27.6%)	0.274	0.46(0.11–1.86)
24.6–29.9 kg/m2	83(26.9%)	12(41.4%)	0.47	1.65(0.43–6.38)
>30 kg/m2	4(1.3%)	6(20.7%)	0.007	12.1(2.00–73.19)

BMI: Body Mass index, TRH: Treatment Resistant Hypertension, AOR: Adjusted Odds Ratio, HTN: Hypertension, CI: Confidence Interval

## Discussions

TRH has become a threaten for health care professionals in management of hypertension, prevention of hypertension related complications, control of BP, and it increased cardiovascular risks and premature death remarkably [32]. To best of our knowledge, this study was the first to study the prevalence of apparent TRH and identify factors associated with resistant hypertension in ambulatory patients in the country and the region. According to our assessment and evaluation, 8.6% of the studied hypertensive patients were resistant for treatment. Furthermore, BMI greater than 30kg/m^2^ and six years and longer duration of hypertension were the factors which predicted TRH.

This study found TRH on 29 ambulatory hypertensive patients, which made the prevalence of resistant hypertension to be 8.6% [CI:0.056–0.116]. A relatively similar prevalence’s were reported in studies conducted in USA (8.9%) [30] and Malaysia (8.8%) [33, 34]. On the contrary, this finding’s treatment resistance was quite higher when it is compared to a study done in china which illustrated a TRH prevalence of 1.9% [35] and Israel with resistant hypertension prevalence of 2.2% [34]. The difference might be occurred because of the study discrepancy between the two studies. The study from china was a cohort study which followed patients for two weeks to declare treatment resistance while in our study data was collected from patients’ medical record retrospectively and TRH was affirmed using the last follow up’s BP measurement. Furthermore, the discrepancy might be due to the antihypertensive regimens utilized variation between the studies. In the study from china patients were given 3 antihypertensive medications then they were assessed whether their BP was controlled or not. Patients were used dihydropyridine calcium antagonist along with an ACEIs or angiotensin receptor blocker (ARB), a beta blocker, or a thiazide diuretic [35]. In our study, patients were taking thiazide diuretics, dihydropyridine calcium channel blockers along with an ACEIs but beta blockers were not used. This regimen difference might considerably depart the prevalence’s of the two setting making Chinese TRH lower than ours.

Our studies prevalence of resistant hypertension was lower as compared to many studies from the globe. A couple of studies found a prevalence of TRH to be 10.3%[13], 12.2%[36], 13.3% [37], 17.7% according to 2008 statement definition and 19.7% according to 2018 statement definition [14], 15%[15], 18.7%[38], 19.1%[37], 24.7%[39]38% [24] and 54% [40]. Moreover, it is lower than TRH prevalence of Cameroon, 11.7% [17], Burkina Faso, 14.6% [18], Lesotho, 14.3% [19] and Algeria, 19.0% [20]. The discrepancy is significant and the variation might be due to the difference in provision of pharmaceutical care and clinical service, variation in availability of antihypertensive medications and patient’s management protocol. In our study majority (66.8%) of the patients were on monotherapy despite uncontrolled BP which showed that medication escalation based on patients BP control status was not aggressive. This low aggressive intervention of pharmaceutical care providers might enable us to discern low prevalence of apparent TRH. The studies with greater prevalence’s are relatively richer than our country. They have better detection ability and experience of treatment resistant, a number of antihypertensive medications options and thus could frequently change the regimen if they wish, better health care provision, and better attitude, knowledge and practice of the their patients towards their medical illness [13–15, 24, 36, 38].

Patients with BMI greater than 30kg/m^2^ were more likely to be resistant for treatment than patients with normal BMI. There are many studies advocating this finding. Studies from USA reported that obesity is a common risk factor for TRH [41, 42]. Increasing prevalence of hypertension and the resistant hypertension has been strongly associated with being overweight or obesity. This might be due to accumulation of aldosterone. Studies showed that aldosterone levels were positively correlated to increasing BMI. Obesity specifically abdominal obesity attributes to excess aldosterone collection which finally result to uncontrolled BP [23].

Patients who were on management and follow-up for 6 years and above were about 4 times more likely to be resistant for antihypertension treatment as compared to patients who were on management for less than 6 years. A similar finding was reported from a study done in Sri Lanka, South Asian origin [37]. With time the probability of up titration of antihypertensive medications also escalate. When antihypertensive medications use inclined, patients will be probably more likely to resist to hypertension treatment. With aging BP control is unsuccessful and most importantly, use of more than 3 antihypertensive drugs also increase [34].

Our study is not immaculate and thus has flaws like many other studies. One thing we used secondary data from medical registries, therefore a number of variables such as diet, salt intake, physical exercise, and restriction of high fat intake were left unstudied. Secondly, the type of resistant was apparent resistant hypertension not true resistant hypertension since patients with white-coat hypertension, improper BP measurement or medication non adherence were not excluded. Thirdly, because the study design was a cross sectional we could not affirm a causal association of the variables and TRH. Fourthly, the study was conducted in a single hospital it is difficult to generalize the result to the general population.

## Conclusions

Apparent TRH is common in the study setting despite low scaling up of antihypertensive medications. Obesity and longer duration of hypertension were associated with apparent TRH. Great emphasis should be rendered to estimate true hypertension resistance prevalence and future studies should descry the effect of aggressive antihypertensive medications escalation on the risks of TRH.

## Supporting information

S1 Fig Patient selection flow chart of hypertensive patients in Mekelle Hospital, 2019. (DOCX)

## List of abbreviations

ACE: Angiotensin Converting Enzyme
ACEIs: Angiotensin Converting Enzyme Inhibitors
ALLHA: Antihypertensive and Lipid‐Lowering Treatment to Prevent Heart Attack Trial
AOR: Adjusted Odds Ratio
aTRH: Apparent Treatment Resistant Hypertension
BMI: Body mass index
BP: Blood pressure
CHF: Congestive Heart Failure
CI: Confidence Interval
COR: Crude Odds Ratio
CVDs: Cardiovascular diseases
DBP: Diastolic blood pressure
DM: Diabetes mellitus
HCT: Hydrochlorothiazide
HTN: Hypertension
RVI: Retroviral Infection
SBP: Systolic blood pressure
SPSS: Statistical Package for Social Science
TRH: Treatment Resistant Hypertension
US: United States
USA: United states of America
