# Supplementary material for: Treatment resistant hypertension among ambulatory hypertensive patients: A cross sectional study
Source: PLoS One. 2020 Apr 28;15(4):e0232254. doi: 10.1371/journal.pone.0232254 (PMC7188288; doi:10.1371/journal.pone.0232254)
Supplement: S1 Fig — (DOCX) [file pone.0232254.s001.docx]

Total hypertensive patients registered in Mekelle hospital=2000

Patients who do not came for follow up during the data collection period=379

Patients with no full record of BP, type of medication, and BMI=336

Patients who came for follow-up during data collection period=1621

Patients exclude via lottery method=423

Patients excluded from study because they have less than 6 months follow up period=359

Total patients analyzed=338

Patients excluded from study because they were on Dietary Approach to Stop Hypertension(DASH)

=165

**Supplementary Figure 1: Patient selection flow chart of hypertensive patients in Mekelle Hospital, 2019.**
